# Engagement in Everyday Activities for Prevention of Stroke: Feasibility of an mHealth-Supported Program for People with TIA

**DOI:** 10.3390/healthcare9080968

**Published:** 2021-07-30

**Authors:** Ann-Helen Patomella, Lisette Farias, Christina Eriksson, Susanne Guidetti, Eric Asaba

**Affiliations:** 1 Department of Neurobiology, Care Sciences and Society, Division of Occupational Therapy, Karolinska Institutet, 14183 Stockholm, Sweden; lisette.farias.vera@ki.se (L.F.); christina.eriksson@ki.se (C.E.); susanne.guidetti@ki.se (S.G.); eric.asaba@ki.se (E.A.); 2 Academic Primary Health Care Center, 11365 Stockholm, Sweden; 3 Unit for Research, Education & Development, Stockholms Sjukhem Foundation, 11219 Stockholm, Sweden

**Keywords:** cardiovascular disease, behavior change, app, stroke, secondary prevention

## Abstract

Most of the risk factors for stroke are modifiable, yet incorporating and sustaining healthy lifestyle habits in daily life that reduce these risk factors is a major challenge. Engaging everyday activities (EEAs) are meaningful activities that are regularly performed that have the potential to contribute to the sustainability of healthy lifestyle habits and reduce risk factors for stroke. The aims of this study were (1) to investigate the feasibility and acceptability of a digitally supported lifestyle program called “Make My Day” (MMD) for people at risk for stroke following a transient ischemic attack, and (2) to describe participants’ stroke risk and lifestyle habits pre- and post-intervention. A multiple case study design using mixed methods was utilized (*n* = 6). Qualitative and self-reported quantitative data were gathered at baseline, post-intervention, and 12 months post-baseline. The results indicate that MMD can support lifestyle change and self-management for persons at risk for stroke following a TIA. The findings indicate a high acceptability and usability of MMD, as well as a demand for digital support provided via a mobile phone application. Self-management with digital support has the potential to increase participation in EEAs for persons at risk for stroke following a TIA.

## 1. Introduction

This paper explores secondary stroke prevention through six case studies included in the Make My Day (MMD) prevention program. MMD combines group sessions and individualized self-monitoring via a mobile phone application (app) to support persons at risk for stroke in incorporating engaging everyday activities (EEAs) into their lifestyle [1]. Major public organizations and government agencies such as the American Heart Association (AHA) and the Swedish National Board of Health and Welfare emphasize the need for preventive programs to address modifiable risk factors (health behaviors) for stroke (i.e., smoking cessation, physical activity, healthy dietary habits and weight reduction) and, if needed, to help prevent unhealthy alcohol consumption [2,3,4].

### 1.1. Engaging Everyday Activities and Lifestyle

Meaningful and purposeful everyday activities combined with moderate-intensity physical activities and a healthy diet have been found to be strongly related to well-being and longevity [5]. One assumption is that when meaningful and purposeful activities are engaging, they have greater potential to be incorporated and maintained in everyday life compared to health-promoting activities in general. Engaging everyday activities (EEAs), when conceptually applied to an intervention, can be situated as a behavioral change technique [6]. The concept of EEAs refers to an individual perception of personal activities that are valuable, meaningful, and purposeful, as well as providing positive feelings and an intense sense of participation. EEAs are activities performed regularly and as part of a person’s life [7]. EEAs can go beyond personal pleasure and can have a higher level of importance due to their meaning for others such as family, friends, or society at large. EEAs are the things that people perform to make life worth living and can contribute to well-being [7,8]: for example, being with family, playing with grandchildren, taking long walks in the forest, or attending dance sessions with others. Studies have shown that promoting EEAs can have positive health impacts on older adults [9,10,11].

EEAs are drawn upon here as a means and goal for changing and sustaining a healthy lifestyle and have the potential to transform a person’s lifestyle and health. In the context of health, lifestyle is mostly associated with indices such as physical activity, eating habits, and the use of or consumption of tobacco and alcohol [12,13]. Broadly, lifestyle encompasses choices, behaviors, and everyday activities that unfold in a sociocultural context [14]. Risk factors for stroke can improve or worsen depending on the balance between various choices, behaviors, and everyday activities, alternatively being impacted by the degree to which there are conditions which influence lifestyle.

Although EEAs can play a key role in incorporating positive lifestyle changes that contribute to reducing risk factors for stroke, there is a need to systematically explore the potential of EEAs in stroke secondary prevention. In this paper, we specifically focus on non-pharmacological and non-surgical stroke secondary prevention, which has been defined as support to improve long-term health and well-being in everyday life, and as a means to reduce the risk of another stroke, by drawing from a spectrum of theoretically informed interventions and educational strategies. Interventions to self-manage modifiable lifestyle risk factors are contextualized and individualized to the capacities, needs, and personally meaningful priorities of individuals with stroke and their families [15].

### 1.2. Transient Ischemic Attack and Risk Factors for Stroke

Transient ischemic attack (TIA) is defined as a transient episode of neurological dysfunction caused by focal brain, spinal cord, or retinal ischemia, without acute infarction, and of a brief duration of <1–2 h [16,17]. Epidemiological studies report incidence rates of first-ever TIA (standardized to the European population) ranging from 25 to 73 per 100,000 inhabitants per year [18]. The risk of a stroke following TIA has been estimated to be 10.5% within the first 90 days, and half of these occurred as early as 48 h post-TIA occurrence [19]. An unhealthy lifestyle is associated with a higher risk for a subsequent stroke and with higher mortality after stroke [20]. Risk factors for TIA and stroke can be classified into three major groups: (a) non-modifiable risk factors such as age, gender, ethnicity, and family history; (b) medically modifiable risk factors such as hypertension, hyperlipidemia, and diabetes; and (c) behaviorally modifiable risk factors such as tobacco, physical activity, and diet, which can be modulated by changes in lifestyle [3]. In addition, medically modifiable risk factors can also improve with changes in lifestyle, e.g., hypertension. The high risks for a stroke associated with a TIA warrant early secondary stroke prevention and, in particular, targeting behaviorally modifiable risk factors [21,22].

### 1.3. Primary Healthcare and Policy

Physicians in primary healthcare face challenges in terms of time constraints to offer non-pharmacological and non-surgical interventions to educate and support patients in their lifestyle changes [23], which suggests a need for interprofessional collaboration with other professionals such as nurses, occupational therapists, nutritionists, psychologists, and physical therapists in lifestyle-based preventive primary healthcare interventions. Although the benefits of a healthy lifestyle are clear [3,4,24], the consistency of implementation and long-term effects of primary healthcare-based lifestyle interventions is not well established [25,26].

Mobile health (mHealth) options have been widely studied during the past 15 years, resting on an idea that technology will enable providers to reach more patients. With almost two billion people currently owning a smartphone, the use of mHealth interventions such as text messaging and apps for smartphones and wireless devices has been increasingly used in, for example, stroke prevention, and now, following the pandemic, there will be even more progress in this field. Thus far, most mHealth interventions, such as smartphone app interventions, are largely unregulated and most are not evidence-based [27]. App-based interventions combined with other strategies such as health education or peer support have been more effective in changing diet or physical activity behaviors as compared to stand-alone app-based interventions [28]. There is an urgent need to develop and test interventions that utilize a combination of strategies to support adherence to healthy lifestyle (behavior) changes (i.e., increased participation in health-promoting EEAs) among people that have suffered a TIA to prevent stroke and TIA recurrence [4]. Increasing literacy about risk factors and promoting individualized programs such as MMD based on personally meaningful priorities could be a way forward to successfully and sustainably incorporating healthy lifestyle patterns into everyday life [29].

This feasibility study [30] of MMD will contribute to adapting an intervention before running a full-scale trial and is particularly useful in exploring the acceptability, recruitment, and delivery of the intervention [31]. The aims of this study were (1) to investigate the feasibility and acceptability of a digitally supported lifestyle program called MMD for people at risk for stroke following a transient ischemic attack, and (2) to describe participants’ stroke risk and lifestyle habits pre- and post-intervention.

## 2. Materials and Methods

### 2.1. Design

This study is a multiple case study [32] with a mixed methods design [33] including both qualitative and quantitative data.

### 2.2. Sample

Inclusion criteria were: (a) age of 55–75; (b) three or more risk factors for stroke scored as high risk according to the stroke risk score card [34] (i.e., smoking, physical inactivity, poor diet, diabetes, high blood pressure, atrial fibrillation, high cholesterol, and family history of stroke); (c) access to either a smartphone or other wireless device; and (d) motivated for a lifestyle change based on self-report. Recruitment was conducted at two major hospitals in the Stockholm urban area via clinicians (recruited two participants) and a TIA register (recruited 15 participants). Seventeen participants (four women) at risk for stroke were identified and contacted, and eight agreed to participate. Among the eight persons who agreed to participate, one woman did not fulfil the criteria of having access to a smartphone or wireless device, and one man had to withdraw due to working full-time (see flowchart). In total, four men and two women participated, and the mean age of the participants was 64.5 (range 59–70). Three of the participants lived alone, and the others lived with a partner or a child. The time since TIA varied from 1 to 15 months (median = 4 months). The level of education varied: one participant with primary education, three with secondary education, and two with a higher education level. The participants’ risk factors at baseline with fictive names are presented in Table 1. George had two high-risk factors but was included since he was medicating for hypertension. Self-reported motivation for lifestyle change [35] was high or moderate.

### 2.3. The Make My Day Prevention Program

The prevention program is evidence-based and theoretically underpinned by behavioral theory [36] and occupational science [1]. It has previously been emphasized that for an intervention to be efficient, it should include three or more behavioral change techniques and support the person to achieve and maintain an active lifestyle [37]. The program enables a reduction in stroke risk factors using four behavioral change strategies: (1) setting realistic goals [38] and sharing experience in a learning environment [39], (2) incorporation of health-promoting EEAs, (3) forming new habits that prompt conscious decisions and healthy choices [40], and (4) use of mHealth technology to increase health literacy and awareness of current habits and increase self-management skills [41].

The program started with an individual meeting (baseline) in which participants set three individual goals concerning their needs and motivation. Goals included regular participation in health-promoting EEAs and change in lifestyle habits, e.g., healthy eating, exercise, and physical activity. Motivational interviewing techniques [42] and the Canadian Occupational Performance Measure [43] were used to help identify areas in which participants were motivated to change. After this individual meeting, the program included five face-to-face group sessions over 5 weeks with a booster session five weeks later, during a total of 10 weeks (see Table 2). Although evidence for a booster session is insufficient, and some studies find insignificant effects [44] while others find reasonable gains from the perspective of sustaining new behaviors [45,46], the booster session was included based on results from earlier phases of designing and testing this intervention [8,47].

Each face-to-face group session had a theme and related activities with the purpose to create awareness of stroke risk factors and current activity patterns. Group dynamics were used to reflect on participant experiences and to discuss the topics related to the sessions’ theme. The sessions were delivered by an interventionist/researcher (AHP and CE) together with a trained health professional (trained during two half-days), i.e., an occupational therapist, a physiotherapist, or a dietician. Each session lasted for 120 min and was held at a healthcare facility in central Stockholm.

#### The Digital Platform

Between face-to-face sessions, a digital mHealth platform (app) was used by participants to monitor their progress toward their goals, including participation in meaningful EEAs. This monitoring consisted of activities such as registering goals and goal fulfilment, and logging EEAs as well as the daily number of steps. The purpose of the app was to support participants’ daily engagement in health-promoting EEAs and facilitate self-reflection on their lifestyle habits by using the app features.

**Figure 1:**
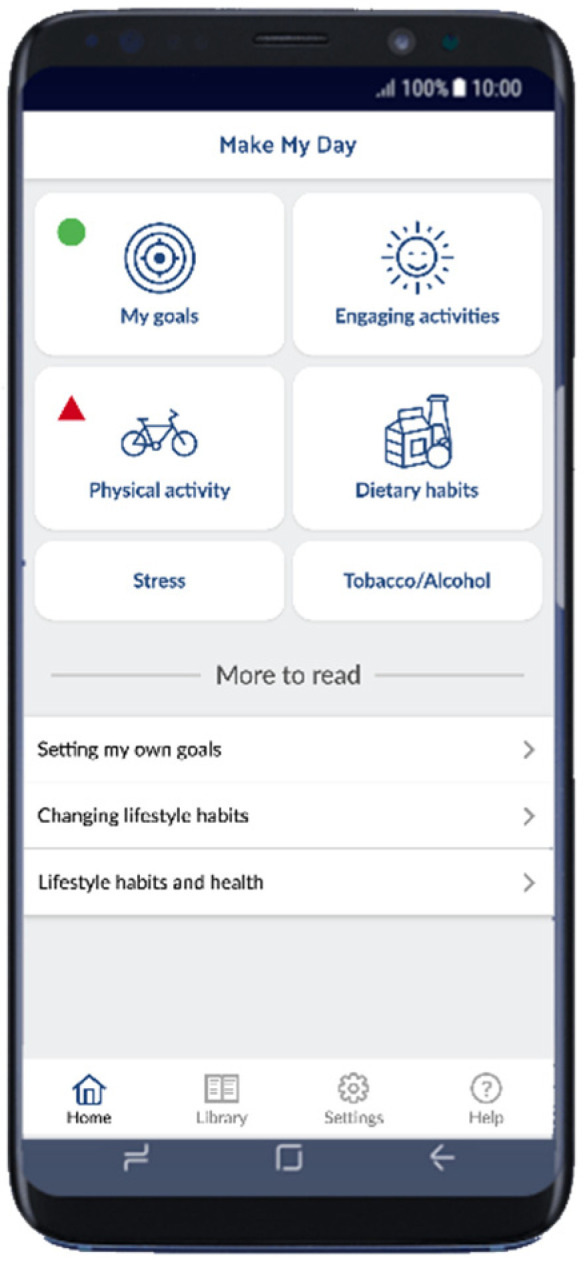
The Make My Day app illustrating the domains and resources.

The app included six domains for registering: (1) Goal achievements (scored as completely, partly, or not achieved today), (2) Physical activity (registering step counts and time in physical activity or exercise), (3) Engaging Everyday Activities (participating in health-promoting EEAs), (4) Tobacco and alcohol use (registering consumption), (5) Stress levels (questions about perceived time pressure), and (6) Dietary habits (registering consumption) (see Figure 1 for illustrations of the domains). The domains were based on behaviorally modifiable risk factors for stroke [3], with the addition of registering EEAs and stress levels. Registrations were shown in visual graphs and plots that informed the participants of the current behaviors. The daily registration in the app was estimated to take about 10 min to complete. The app also includes a library with resources such as films, information, definitions of concepts, and facts of relevance (see Figure 1 “More to read”). The app was developed in close collaboration with ScientificMed Tech AB (http://www.scientificmed.com, (accessed on 30 July 2021)).

### 2.4. Data Collection

Data were collected at baseline, during the intervention, during the post-prevention program, and 12 months post-baseline. Data collection at baseline and during the intervention was performed by two of the authors (C.E. and A.-H.P.), and follow-up data were collected by a research assistant who was not involved in delivering the intervention. At baseline, participant characteristics were collected including outcome measures, motivation for change, and self-perceived stroke risk.

#### 2.4.1. Feasibility Measures

The focus was to investigate the feasibility aspects of acceptability and usability, and data were collected during the whole study process. Feasibility data were collected from participants and interventionists. Acceptability examines if the intervention was satisfactory and attractive for the participants and how they reacted to the content and delivery of the intervention [31]. Data about satisfaction and usability of the app were collected through telephone interviews with the participants at risk for stroke. Interviews were conducted (by AHP and CE) during the program (week 5). Open-ended questions were asked, e.g., “What are your thoughts about the app?”, and “How did the goal-setting domain work for you?”. Questions about the general practicality of the app and for each domain were asked. Data on the usability of the app were also collected from the daily registrations and goal achievements of the participants. Satisfaction with the whole program was examined by using semi-structured interviews post-intervention and at 12 months follow-up with the participants at risk for stroke.

Interventionists and healthcare professionals reflected on the feasibility aspects after each face-to-face group session, and these reflections were recorded. Individual semi-structured interviews were conducted with interventionists following an intervention to include their experiences with the program’s content and delivery. Interviews with interventionists were conducted by a research assistant.

#### 2.4.2. Outcome Measures

The Stroke Risk Score card [34] was used to measure an individual’s stroke risk. The stroke risk score card entails three columns in different colors: red, yellow, and green. Red is high risk, yellow is caution, and green is low risk. The results are divided into high risk: ≥3 red points; caution: 4–6 yellow scores; and low risk: 6–8 green scores.

The Swedish Lifestyle Habits survey is based on guidelines for prevention by the National Board of Health and Welfare in Sweden. The survey includes questions in four domains: physical activity, alcohol consumption, tobacco use, and dietary intake. It complements the stroke risk score card with details on, for example, how many cigarettes the person smokes per day, and minutes/week performing physical activity and exercise.

EQ-5D was used to measure quality of life [48], which comprises five dimensions: mobility, self-care, usual activities, pain/discomfort, and anxiety/depression. Each dimension has three levels: no problems, moderate problems, and severe problems. In addition, a VAS (visual analogue scale) (1–100) was used by participants to mark their current state of health: 1 = worst imaginable health, and 100 = best imaginable health.

A global rating of change scale [49] was used to capture change post-intervention (follow-up) in comparison to baseline in five lifestyle domains: general health, dietary habits, physical exercise, participation in engaging everyday activities, and healthy activity patterns, as designed by the research team. The changes are scored on a scale from −5 to +5, where −5 = extreme decrease in relation to my health, 0 = no change, and +5 = extreme increase in relation to my health.

### 2.5. Data Analysis

Descriptive statistics and qualitative approaches were used to explore the data.

#### 2.5.1. Analysis of Feasibility Data

The usability of the app was investigated by descriptively analyzing the telephone interviews with the participants at risk and by calculating the actual use of the mobile phone app. Calculations were based on registration in each domain in the app and presented in percentages as divided by days within the program. Goal achievement was investigated to add a perspective of usability and analyzed based on app registrations.

Interviews with persons that had a TIA and interventionists and healthcare professionals were analyzed using a qualitative constant comparative approach [50].

#### 2.5.2. Within-Case Analysis of Outcomes and Goal Achievement

Baseline and follow-up data were used to describe and visualize the participants’ change progress in relation to stroke risk factors and health factors. The participants’ goal achievements (based on data from app registrations) during the program period were calculated based on goals achieved (as registered in the app). Missing data, i.e., no registration, were defined as the times that a participant did not fulfil the planned goals for a day.

## 3. Results

### 3.1. Feasibility of the Program and App

During the face-to-face group sessions, the attendance was generally high. All participants attended sessions 1 and 3; for sessions 2, 4, and 6, one participant was absent; and during session 5, two participants were absent. Absence was spread among the participants and was related to reasons such as traveling or sickness (e.g., having a cold).

#### 3.1.1. Acceptability of the App

In general, all participants at risk for stroke were satisfied with the mobile phone app. At the start of the program, one of the participants reported difficulties using the app and was provided with extra instructions for its use by an interventionist/researcher. The participants emphasized that the features of the app were suitable and fun. John said, “*Overall a good app, suits me perfectly.*” Mona reported, “*It was fun to use the app*”. Moreover, all participants agreed that it was useful to be able to register and keep track of their goal achievement. However, some participants mentioned that it was difficult to remember their selected goals, as the app only prompts them: “Did you reach your first goal?” This feature meant that participants needed to revisit their notes to revise and remember their goals. According to five of the six participants, it was difficult to understand the concept of EEAs as it was perceived as abstract, and therefore registering their level of participation and self-efficacy in EEAs was perceived as challenging. All participants felt that the domains related to stress, food intake, and tobacco and alcohol use were concrete and easy to register. When being asked if they would continue using the app, all participants responded that they wanted to continue using it, indicating a demand for the app. In summary, based on the extent to which participants stated that the app met their needs for tracking and achieving their goals, the acceptability of the app was considered high.

#### 3.1.2. Usability of the App

The usability of the app is based on the rate of participants’ daily registrations of goal achievement and lifestyle habits (see Table 3). Based on the average across all participants and domains in the app, the rate of daily registrations was 81.8%, which was considered to represent the high usability of the app. With 54.8% of daily registrations, Sam had the least registrations in the app (as he did not register any goal achievements), and Tina had the highest compliance with registering daily, with 99.4% of the days registered over the 10 weeks.

All participants set three goals related to lifestyle change at baseline, based on their motivation, and to EEAs (everyday activities that they perceived as engaging and important) (see Table 4). These goals were revised during the program through discussion between participants and interventionists; in three of the cases, the goals were revised to make them more relevant to the participants. Mona had an initial goal to go to the gym once a week, but the goal did not engage her. Consequently, she had difficulties motivating herself to comply, and a couple of weeks later, she revised this goal with support and changed it to go dancing once a week instead (i.e., she loved dancing and used to go dancing before). Although Mona’s goal was once a week, she sometimes engaged in dancing two–three times per week.

The usability from the perspective of registering goal achievement varied among the participants. Tina’s and John’s mean registered goal achievements during the period were high, above 70% (see Table 4 in brackets), meaning that they fulfilled their goals most of the days during the intervention period. Mona had a low mean percentage of goal achievements during this period. Sam had no valid registrations of goal achievements. Sam did register in other domains in the app, and the reason he did not register goals was reported to be because he lost his folder with his written goals.

#### 3.1.3. Acceptability of the Prevention Program as a Whole

##### Experiences of Participants at Risk

The participants with TIA described the program as very beneficial and valuable to support and sustain lifestyle changes. The program was described as “Surprisingly engaging” (John), social, and fun. The group format was highly appreciated and the possibility to meet and reflect on one’s habits was inspiring. The interventionists were described as knowledgeable, inclusive, and open to discussing individual experiences. Follow-ups (post-intervention, 6 months, and 12 months) were experienced as supportive and valuable to sustain one’s new healthy habits and to reflect on what one had learned in the program. Follow-up within healthcare after a TIA varies depending on the hospital and geographic area where a person is registered in primary healthcare. For Mona, who did not receive follow-up from her primary healthcare provider, the intervention served as follow-up and was therefore experienced as extra important.

The app was experienced as an important part of the program. The app was described as showing the participants a picture of one’s habits and led to awareness about what one had performed during a day and that a lot of time was spent in sedentary activities. Habits could be tracked over time, which lead to reflections that Sam called an “eye-opener”. Registration in the app was experienced differently among the participants: some experienced it as easy to complete, others as difficult. Difficulties were related to scoring experiences with an actual number (to be able to assign a figure to an experience), remembering to register each day, and experiences of uniformity while registering (boredom in carrying out the task).

##### Experiences of Interventionists

The interventionists were involved in the enrollment phase of the study and emphasized the difficulties to find and recruit participants at the clinics; therefore, the recruitment period was prolonged for another four weeks. The most valued part of the prevention program, as expressed by the interventionists, were the group dynamics during the face-to-face group sessions, i.e., how the participants naturally created a social context and a culture of sharing their experiences. Although the program was structured around pre-set themes, the participants’ experiences were given attention. The program structure was experienced as holistic regarding the prevention of stroke, focusing on the everyday life situation of the participants and creating the content based on their needs. The sessions lasted for 2 h, but interventionists expressed difficulties to keep each session at 2 h because participants had a lot to share. Although the participants represented diversity in terms of their demographic background, the interventionists and healthcare professionals found that the participants found ways to relate to each other, creating a supportive group dynamic in terms of peer support. The interventionists found that the engagement in the program’s activities was generally high among the participants. In summary, the interventionists and healthcare professionals stated that the participants were always keen to discuss and reflect on each theme’s content.

#### 3.2. Risk for Stroke, Life Satisfaction, and Change Pre–Post-Intervention

Overall, the participants’ modifiable stroke risk factors decreased at follow-up, and only one participant kept the initial three high-risk factors for stroke at follow-up. The other participants decreased their risk factors (see Table 5). Concerning alcohol consumption, none were in a risk category, and no change was recorded between baseline and follow-ups. The participants reported a somewhat higher quality of life at follow-up compared to baseline. At 12 months follow-up, lifestyle habits were sustained, except for physical exercise, which was reduced in two participants that were performing regular exercise at follow-up, but none at 12 months follow-up.

**Figure 2:**
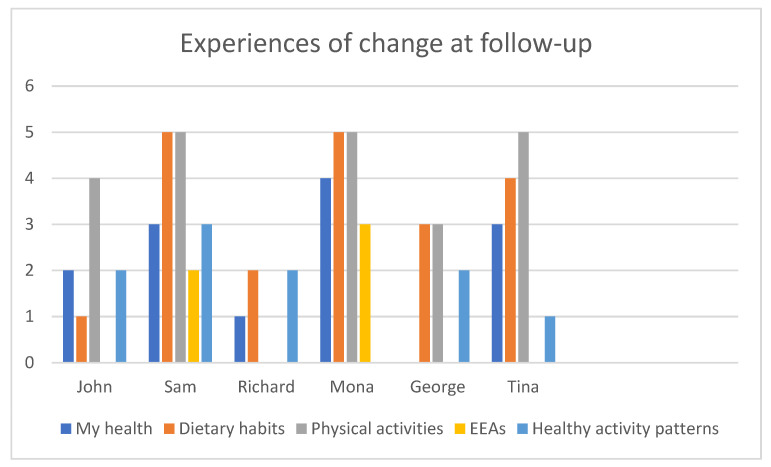
Experiences of change at follow-up (post-intervention).

The participants scored their subjective change post-intervention (follow-up) within five domains: general health, dietary habits, physical activity, participation in EEAs, and healthy activity patterns. Physical activity and dietary habits were experienced as having increased the most (see Figure 2). General health increased in five of the six participants as the activity patterns also became healthier (Figure 2).

**Figure 3:**
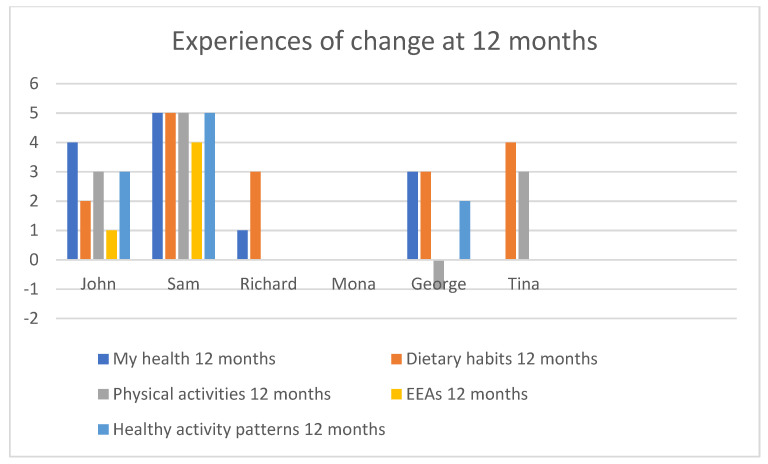
Experiences of change at 12 months follow-up.

At 12 months follow-up, John, Sam, and Richard sustained most of the changes achieved at follow-up (compared to baseline), while George decreased his physical activity, and Tina’s general health and healthy activity patterns were decreased (see Figure 3). The experienced change in dietary habits since baseline was maintained for all participants at 12 months.

## 4. Discussions

This study examined the feasibility of a new lifestyle program for people at risk for stroke following a TIA by using a combination of face-to-face group sessions and mHealth support (a mobile phone app) over 10 weeks. The findings suggest that the program including the use of mHealth has the potential to support individuals in self-managing modifiable risk factors by increasing their level of physical activity and healthy nutrition.

The findings indicate participants’ high satisfaction with the program and the app, suggesting high acceptability [51]. The participants appreciated the app and demonstrated high and moderate usability in relation to daily registration and reporting, except for one participant. These results are in line with previous studies that emphasize the potential of mHealth in cardiovascular event prevention for delivering useful and accessible support [27]. It has been indicated that mHealth can lead to a greater effect in comparison to information only [52,53]. However, mHealth services and apps still show gaps between conceptual understandings and translation into real life [27], which was also the case for the app used in this program. Participants expressed that the concept of EEAs was difficult to understand and therefore to register in relation to participation and self-efficacy. This indicates that the concept needs to be formulated and experienced by the participants themselves before starting to use the app and registering. This was confirmed by four of the participants that did not report a self-experienced change for this item at follow-up.

The program and face-to-face sessions were highly appreciated by the participants and suggest that a blended program including both group meetings and mHealth support could be beneficial in a lifestyle change process [54,55]. It has also been suggested that relatives should be involved in the lifestyle change process [29,56] and that a prevention program that involves family members could benefit participants in improving their health in a more sustainable manner. The inclusion of family members is a feasible way to further develop the MMD program.

The participants in this study showed a self-experienced change in lifestyle and health, and the change was more evident at follow-up than at 12 months. At 12 months, regular physical activity was a challenge, but dietary habits were still maintained. In keeping with prior studies, moderate physical improvements can be found immediately following primary healthcare prevention, but sustainability is a challenge [25,57]. It has been suggested that there is a need for more booster strategies over longer periods to maintain physical activity levels [58]. To prevent stroke and TIA, a program that provides guidance on how to obtain and maintain an active lifestyle in combination with guidance on medication, smoking, alcohol intake, and dietary habits is recommended [37]. The MMD intervention includes these parts and uses EEAs to implement lifestyle change with the support of mHealth for self-managing risk factors for stroke.

### 4.1. Methodological Limitations

The sample mainly consisted of men, although the qualitative data highlight all participants’ voices including the two participating women. No differences between sexes could be found in the small sample size of this feasibility study, although this was not the aim.

A limitation of the app was registering goal achievement. It was not possible for the participants to see or read the goals (within the app) that they created at baseline, resulting in participants having trouble remembering their goals and reducing compliance (one participant did not register his goal achievement). Although all participants received their goals on paper in a folder, there was a risk for losing the folder. From this perspective, the mHealth tool can have some advantages and be more sustainable if acceptable for the person. It is also important that the app allows for registering goals on a daily and weekly basis. The current version only allowed registration on a daily basis even though weekly goals were also relevant (e.g., go dancing once a week), which was a limitation in the app.

The results of the study are mainly based on self-reported data and on a small sample and should be treated cautiously. The change scores are based on experiences post-intervention and could be biased due to difficulties in the lack of insight or remembering one’s previous health status. The participants’ physical fitness was not tested before or after the intervention. Based on feedback on the exercise segment of this trial, there is a need to compare self-reported physical activity levels and physical fitness before drawing conclusions about the participants’ physical health as well as integrating a way to provide the just-right physical challenge during the intervention. The outcomes of this study cannot be generalized to a larger population, and future studies should include a higher number of participants and a randomized control group. Some advantages of this feasibility study are that it was time- and cost-effective [51] and that future studies can use the results to refine and advance methods.

### 4.2. Clinical Implications

Cardiovascular events including TIA and stroke are the leading cause of death in the world. In Sweden, there is a lack of structured methods and programs in primary healthcare to help patients that had a TIA achieve a healthy lifestyle, making this study important for the development of such interventions. The results from this study indicate that mHealth-supported interventions can be suitable in non-pharmacological and non-surgical stroke secondary prevention. Since this study suggests that there is demand and acceptability among participants for such a program, there is a need to further develop mHealth-supported interventions in this area. Future research will need to continue exploring the optimal combination of app features and behavior change strategies to maximize motivation and intervention efficacy among persons at risk for stroke.

## 5. Conclusions

The findings from this study suggest that the MMD program was highly accepted by the participants including the interventionists and primary healthcare professionals, that lifestyle change goals could be achieved, and that health could be improved. Although physical activity goals were hard to sustain over time (12 months follow-up), dietary goals were maintained. The mHealth service used in the program (mobile phone app) was accepted and usable for the participants.

## Figures and Tables

**Table 1:** Participant characteristics at baseline.

	John	Sam	Richard	Mona	George	Tina
High stroke risk factors according to the stroke risk score card [34]						
Hypertension > 140/90 (Medicating Y/N)	Y (Y)	Y (Y)	Y (Y)	C (Y)	C (Y)	Y (Y)
Overweight ^a^ (BMI)	Y (31)	Y (27)	Y (29)	Y (27)	Y (26)	Y (28)
Atrial fibrillation	N	N	N	N	N	N
Smoker	Y	N	N	N	N	N
Hypolipidemia	N	Y	N	N	N	N
Diabetes	N	N	N	N	N	N
Irregular physical exercise (self-reported aerobic exercise minutes/week ^b^)	Y (80)	Y (60)	Y (180)	Y (180)	Y (0)	Y (0)
Family history of stroke	Y	Not sure	Y	Y	N	N
No. of high-risk factors according to the stroke risk score card [34]	5	4	4	3	2 *	3
Self-perceived stroke risk (1–10)	6	4	Missing data	6	1	5
Motivation for change	High	High	Moderate	High	High	High
EQ5D − VAS 0 = worst imaginable health 100 = best imaginable health	78	50	60	80	75	99

Y = yes (high risk), C = caution, N = no (low risk). * The person was included since he was medicating for hypertension and considered having a high blood pressure as a potential risk factor. ^a^ Body mass index ≥ 25. ^b^ As reported in the Swedish Lifestyle Habits survey.

**Table 2:** Themes of the prevention program’s face-to-face sessions.

1: Risk for stroke and engaging everyday activities
2: Physical activity
3: Dietary habits
4: Activity balance and stress
5: Sustainable health and goal setting
6: Booster session: Identity, resources, and self-management

**Table 3:** Usability of the app in percentages of use/daily registrations.

Participant	Goal Achievement (%)	Participation in EEAs (%)	Physical Activity (%)	Dietary Habits (%)	Stress (%)	Total (%)
John	97	96	97	99	97	97.2
Sam	0	59	87	66	62	54.8
Richard	54	79	87	89	66	75
Mona	73	73	73	73	73	73
George	94	90	90	94	90	91.6
Tina	100	97	100	100	100	99.4
Mean	69.7	82.3	89	86.8	81.3	81.8

Tobacco use is not presented since only one participant used tobacco.

**Table 4:** Lifestyle change goals as set by the participants at baseline (in parenthesis; the new goals as revised later in the program). Goal achievement as registered on a daily basis in percentage.

Participant	Goal 1 (Goal Achievement in %)	Goal 2 (Goal Achievement in %)	Goal 3 (Goal Achievement in %)
John	Keep a healthy weight (76%)	Daily light exercise, i.e., walking 6 K per day (56%)	Swimming 5 times a week (85%)
Sam	Implement new sleeping routines—go to bed at latest 11 p.m. (no valid number)	Eat vegetables with each meal (no valid number)	Physical exercise 3 times a week (no valid number)
Richard	Eat vegetables with each supper (65%)	Light exercise of at least 20 min/day (35%)	Reduce the intake of snacks and cakes (69%)
Mona	20 min daily walking (11%)	Go to the gym once a week (dancing once a week) (44%)	Eat at least two portions of vegetables per day (48%)
George	Go to the gym twice a week (55%)	Eat more fruits (taking lunch walks twice a week) (35%)	Eat more vegetables (42%)
Tina	Walk 10 K per week (running 3 times/week) (76%)	Eat at least one fruit per day (96%)	Eat at least one portion of vegetables/day (92%)

**Table 5:** Factors affecting health, at baseline, follow-up, and 12 months follow-up.

Factors Effecting Health	Baseline (*n* = 6)	Follow-Up (*n* = 6)	12 Months Follow-Up (*n* = 5)
Stroke risk factors			
No. of participants with hypertension > 140/90 (medicating)	4 (6)	2 (6)	1 (5 ^b^)
No. of participants with overweight ^a^	6	5	4
No. of participants with atrial fibrillation	0	0	0
No. of smokers	1	1	1
No. of participants with hypolipidemia	1	1	1
No. of participants with diabetes	0	0	0
No. of participants with irregular physical exercise ^c^	6	1	3
Median number of high-risk factors (range)	3.5 (2–5)	1 (0–3)	1 (0–2)
Quality of life (EQ-5D-VAS 0–100): mean; SD	73.7; 17.0	88.8; 11.7	82.0; 14.8

^a^ Body mass index ≥ 25. ^b^ One of the participants dropped out at 12 months follow-up due to illness (not stroke-related). ^c^ Irregular was defined as less than 75 min vigorous-intensity physical exercise per week [3].

## Data Availability

The data presented in this study are available on request from the corresponding author.
